# Angiosperm flora on the páramos of northwestern Colombia: diversity and affinities

**DOI:** 10.3897/phytokeys.70.8609

**Published:** 2016-09-30

**Authors:** Fernando Alzate-Guarín, Jhon Steven Murillo-Serna

**Affiliations:** 1 Instituto de Biología, Herbario HUA, Universidad de Antioquia. Calle 67 Nº 53-108. Medellín, Colombia

**Keywords:** Angiosperms, Andes, Antioquia, páramos, Colombia

## Abstract

Páramos are high-elevation isolated ecosystems in the Andes characterized by specific flora. This flora includes a number of endemic species and some taxa phylogenetically related to temperate lineages (van der Hammen and Cleef 1986). There are six páramo units or complexes in the Department of Antioquia, located in northwestern Colombia. For five years, we conducted botanic explorations in order to quantify the richness of angiosperm flora in these units. We estimate the richness of angiosperms in these páramos at 693 species, 277 genera, and 86 families, which represent almost 10% of the floral diversity in Antioquia, but contained in only 0.7% of its area. We found that Frontino-Urrao is the most species-rich páramo with 465 species from 225 genera. Our results show that the most diverse angiosperm families of the páramos of Antioquia are Asteraceae, Orchidaceae, Melastomataceae, and Poaceae, which together represent 245 species. Groupings between páramos by Sørensen’s similarity index show that the complexes of the Central Andes Cordillera form a cluster of greater affinity than Páramos from other regions. Of the species found, 80 have a CITES or IUCN diagnosis. The expeditions allowed the identification of 21 species not previously registered in Antioquia and a considerable number of endemisms (35 species), further proof of the high plant diversity in these ecosystems.

## Introduction

The páramo ecosystem has been defined in several ways with differing delimitation methods. One of such definitions (Cuatrecasas 1958) considers the páramo as open high-elevation areas characterized by particular vegetation, with the high-Andean forest as the lower limit and the permanent snowcap as the upper limit. Based on altitude and vegetation structure, Cuatrecasas (1968) proposed a subdivision of the páramo into subpáramo, páramo, and superpáramo. Sklenár et al. (2005) define the páramo as intertropical high-mountain ecosystems, located between the continuous band of forest and the upper limit of the permanent snowcap where vegetation can still be found.

Average incident temperatures in páramos range between 3°C and 9°C, with highly marked diurnal fluctuation of up to 20°C. Rainfall ranges between 700 mm and 5000 mm per year, with relative humidity between 80% and 98%. Generally, the lower limit of páramos is defined at about 3000 m.a.s.l. of altitude (Morales-Rivas et al. 2007). Biophysical criteria, such as climate, altitude, soil, biodiversity, and endemisms help establish the limits between forest and páramo (Van der Hammen and Otero 2007).

Colombian páramos are organized under districts and complexes (Sarmiento et al. 2013) as a way to group natural areas that are in many cases divided by geographic accidents or anthropogenic perturbation. In Colombia there are 39 páramo complexes, 20 located on the Eastern Cordillera, 11 on the Central Cordillera, 7 on the Western Cordillera, and 1 on the Sierra Nevada de Santa Marta (Sarmiento et al. 2013). In Colombia, much of the páramo is located in special conservation zones; however, páramo regions are still threatened by the effects of global warming, the expansion of agricultural areas, and, in the particular case of Colombia, the expansion of mining activities (Benavides 2013).

The Department of Antioquia, located in northwestern Colombia, covers an area of 63,612 km^2^, comparable to the areas of countries such as Costa Rica (51,100 km^2^) or Sri Lanka (65,610 km^2^). Antioquia has six páramo complexes, three of them situated in the Western Andes Cordillera (Farallones de Citará, Frontino-Urrao, and Paramillo) and three in the Central Andes Cordillera (Belmira, Valle de Aburrá, and Sonsón). Páramo regions in Antioquia, including new subdivision proposals made by the Alexander von Humboldt Institute for Biological Research (Sarmiento et al. 2013), currently cover an area of about 46,000 ha, representing 0.7% of Antioquia’s surface area.

Some efforts have been undertaken to quantify the diversity of flora in Colombian páramos; a recent publication by Marín and Parra (2015) stands out among such efforts. Their work lists a total of 658 plant species for Colombian páramos, a figure that is evidently quite far from the actual diversity found for the páramos in the country. Some detailed inventories of páramo flora have been published for areas such as Chingaza (Madriñán 2012), Chisacá (Pedraza-Peñalosa et al. 2001, 2004), and Sonsón (Alzate et al. 2016), among others. Such inventories have allowed the development of more detailed analyses with regards to the evolution and classification of these areas. Miranda et al. (2002) defined the areas of endemism for Colombian páramos by means of Parsimony Analysis of Endemicity  (PAE). Madriñan et al. (2013) inferred speciation rates of some páramo lineages and found that this ecosystem presents the highest speciation rates known for angiosperms. Londoño et al. (2014) proposed floristic and biogeographic affinities for 30 Colombian páramos by using PAE and Jaccard’s similarity index.

In this project we aim to document and evaluate the conservation status of angiosperm species found in the páramos of Antioquia. We also present a floristic affinity analysis based on taxonomic inventories for the six páramos of Antioquia.

## Methods

**Figure 1.**
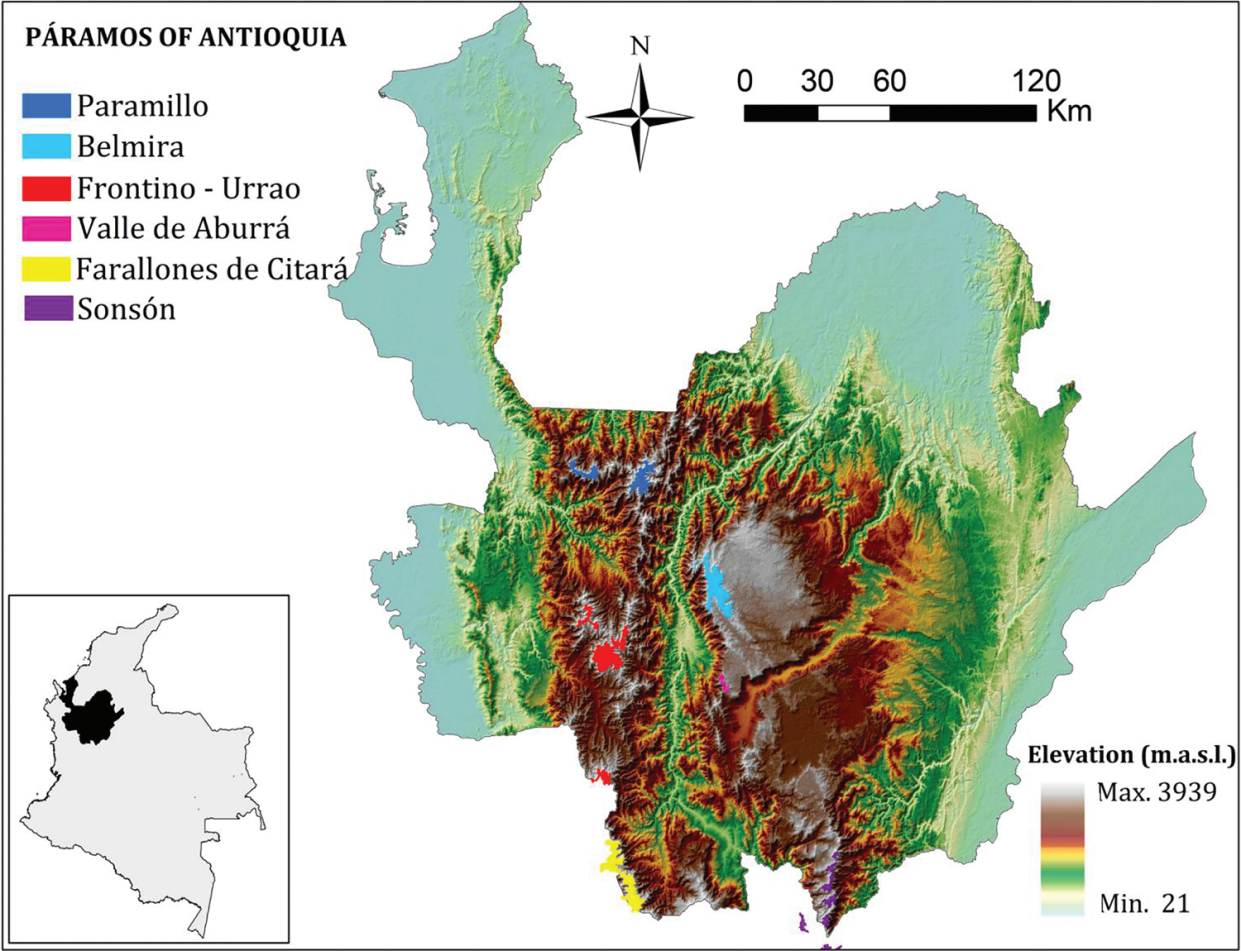
Geographic location of the páramo complexes of Antioquia.

Between 2010 and 2015 we conducted botanical explorations in five of the six páramo complexes in the Department of Antioquia to determine the diversity of Angiosperms in Antioquia’s páramo complexes (Table 1). Páramo areas in Antioquia are located in the Central and Western Andes Cordilleras between 2800 and 3969 m of altitude (Arias 2011). We also included a small azonal páramo found at 2600 m that belongs to the Belmira complex (Fig. 1). We collected in different periods and climatic seasons in order to find a higher number of plants in bloom or with fruits. We thoroughly documented Angiosperm specimens collected during the explorations with photographs to illustrate the flora on a web page hosted by the Missouri Botanical Garden. Plant material was processed at the HUA and MO herbariums, where the exsiccata were deposited. We supplemented the species inventory generated from this fieldwork with data from the Flora de Antioquia project, carried out by the Universidad de Antioquia and the Missouri Botanical Garden, available online at http://tropicos.org/Project/CV.

**Table 1.** Inventory of Angiosperms for each of the six páramo complexes of Antioquia.

Páramo complex	Extension (ha)*	Families	Genera	Species
Belmira	10.622	62	146	257
Farallones de Citará	11.233	59	112	174
Frontino-Urrao	15.396	79	229	460
Paramillo	1.550	33	68	98
Sonsón	*3.389	61	140	229
Valle de Aburrá	870	59	135	234

* Area within the Department of Antioquia.

We constructed similarity dendrograms among the six páramo complexes using Sørensen’s similarity index (Sørensen 1948), a measure of the number of species shared between two sites compared to the total number of species unique to each site alone. This method was selected because it requires only data on taxa presence/ absence rather than abundance indices. We created three presence/absence matrices using taxa (family, genus, and species) as codified characters with 1/0 for each geographic area of the 6 páramo units in Antioquia. We generated a hypothetical zone where all taxa are absent to root the dendrogram. The analysis was carried out with the PAST3.x software package (Hammer et al. 2001) using the UPGMA algorithm and Sørensen’s similarity index to evaluate floristic affinities between páramo units. We repeated the analyses excluding the páramos with low sampling in order to observe the possible effects of low-sampled areas. The information generated in this project is being published in parallel on the web portal of the Missouri Botanical Garden at http://tropicos.org/Project/Paramos.

## Results

We identified 693 Angiosperm species from 277 genera and 86 families in the six páramo complexes of Antioquia (Table 1). These species represent about 10% of the Angiosperm flora reported for the Department of Antioquia (Idárraga et al. 2011). Our results indicate that Frontino-Urrao is the páramo with the highest richness of angiosperm plant species, genera, and families (460, 229, and 79 respectively). The lowest diversity was found in Paramillo, with only 98 species from 33 families.

Of the 86 families present in the páramos of Antioquia, more than 30 are represented by 6 or more species. Asteraceae and Orchidaceae are the most diverse in species number, each represented by 84 species. (Table 2). With regards to genera, 16 plant families had 5 or more genera in the sampled páramos, with Asteraceae (33 genera) and Orchidaceae (23 genera) being the most diverse families in number of genera. About 20% of the genera and 25% of the species correspond to the Asteraceae and Orchidaceae (Table 2). Of the Angiosperm flora found in the páramos of Antioquia, 35 species are endemic, which represents 5.3% of the total flora registered. Six of the endemic species of the páramos of Antioquia belong to Bromeliaceae, five to Asteraceae, and four to Orchidaceae.

**Table 2.** Number of genera and species with their percentages for the 20 most diverse Angiosperm families in the páramos of Antioquia.

Family	Genera	Percentage	Species	Percentage
ASTERACEAE	33	11.91	84	12.12
ORCHIDACEAE	23	8.30	84	12.12
POACEAE	20	7.22	38	5.48
MELASTOMATACEAE	13	4.69	39	5.63
ERICACEAE	11	3.97	30	4.33
RUBIACEAE	8	2.89	20	2.89
CYPERACEAE	8	2.89	18	2.60
ROSACEAE	7	2.53	25	3.61
LAMIACEAE	7	2.53	9	1.30
BROMELIACEAE	6	2.17	29	4.18
SOLANACEAE	6	2.17	13	1.88
APIACEAE	6	2.17	9	1.30
GESNERIACEAE	6	2.17	8	1.15
CARYOPHYLLACEAE	5	1.81	7	1.01
GENTIANACEAE	5	1.81	7	1.01
PLANTAGINACEAE	5	1.81	6	0.87
LORANTHACEAE	5	1.81	5	0.72
CAMPANULACEAE	4	1.44	15	2.16
PRIMULACEAE	4	1.44	9	1.30
BORAGINACEAE	4	1.44	4	0.58

## Description of the páramo complexes of Antioquia

**Belmira**: Located north of the Central Cordillera in the Santa Rosa altiplano. This páramo covers altitudes ranging from 3,000 to 3,340 m.a.s.l. and is one of the largest páramo regions in Antioquia (Sarmiento et al. 2013). We registered 16 species of Bromeliaceae in this páramo, the highest number of species of this family registered in all the páramos studied. In this work, 59 new records of species are described for the Belmira páramo.

**Farallones de Citará**: Located in the Western Cordillera in the southwestern part of Antioquia. It covers an area of 2,030 ha between 3,350 and 3,940 m.a.s.l., but a proposal has been made to include a 11,233 ha extension (Sarmiento et al. 2013). This páramo is highly diverse in species of Melastomataceae and Asteraceae. In this study, 118 species are newly reported for Farallones de Citará.

**Frontino-Urrao**: Located to the southwest of the Department of Antioquia, to the north of the Western Cordillera. It has a large number of wetlands, and its altitudinal range goes from 3,200 to 3,970 m.a.s.l. (Arias 2011). The high diversity of Asteraceae is notable, with 59 species representing 12.8% of the species found in this páramo. Frontino-Urrao is home to vast populations of two *Espeletia* species that are endemic to the region: *Espeletia frontinoensis* Cuatrec. and *Espeletia praefrontina* Cuatrec. Our research revealed 55 new records of Angiosperms for this complex.

**Paramillo**: Located on the northern extreme of the Western Cordillera inside the Natural National Park Nudo de Paramillo. This páramo ranges from 3,300 to 3,720 m.a.s.l. (Sarmiento et al. 2013). Very few explorations have been carried out in this páramo because of difficulties posed to access it. Because of this, there was limited previous knowledge of Angiosperm diversity in Paramillo. The current inventory shows high diversity of species in Melastomataceae and Orobanchaceae. This is likely the result of low sampling, although these two families are known to be diverse in the páramo, especially Melastomataceae.

**Sonsón**: Located in the southeast of Antioquia, in the Central Cordillera. Its highest altitude reaches 3,363 m.a.s.l., and despite the fact that its total extension in the Department is 3,389 ha, only a very reduced area has páramo vegetation cover. Sonsón was only acknowledged as a páramo in 2009, because its small extension did not favor its delimitation (Alzate et al. 2016b). Bromeliaceae and Melastomataceae are outstanding for their diversity in this páramo, with 18 and 21 species respectively. In this project, we registered 30 species of Angiosperms not previously reported for the complex.

**Valle de Aburrá**: Found on the western part of the homonymous valley, it is composed of two steep hills that reach 2,900 to 3,175 m.a.s.l. Human settlements have greatly transformed this páramo and led to ecosystem deterioration and decrease of natural vegetation. Rosaceae (12 spp), Solanaceae (9 spp), and Piperaceae (7 spp) are particularly diverse in this complex. Explorations in this zone registered 33 species not previously reported.

**Figure 2.**
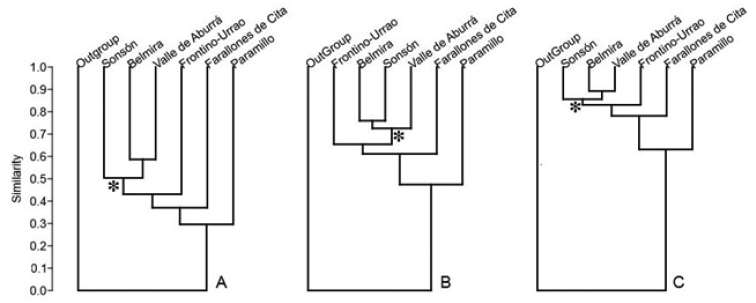
Similarity dendrograms for the six páramo complexes in Antioquia using Angiosperm composition **A** Built using species composition **B** genera composition, and **C** family composition. Asterisk* denotes the node for páramos of Central Cordillera.

Paramos in the Central and Western Cordilleras form separate clusters based on Sørensen’s similarity index when this index is calculated at the species, genus, and family levels (Fig. 2). Of the Western Cordillera, the páramo that is more similar to the ones found in the Central Cordillera is Frontino-Urrao, while the least similar is the Paramillo páramo.

## Discussion

This work documents the occurrence of high plant diversity in the páramos of Antioquia, which represents about 10% of the Angiosperm flora known for this Department. In Colombia there are about 238 (Rangel-Churio 2015) of the 425 Angiosperms families recognized worldwide by Reveal and Chase (2011). The páramos of Antioquia have representatives of 20% of the total families in the world and around 37% of the ones reported for Colombia. Out of the 3431 vascular species reported for páramos by Luteyn and Churchill (1999), Antioquia has around 20%, but only taking into account Angiosperms; if Monilophythos and Lycophytos diversity is included, this percentage could increase to a much higher value.

Most of the plant diversity in the páramos of the Department is found in the Western Cordillera, especially in the Frontino-Urrao páramo. This high diversity is comparable to the diversity of páramos such as Sumapaz or the Nevados (Londoño et al. 2014). However, Frontino-Urrao is much smaller than these páramos; while the páramo of Frontino-Urrao is only 13,921 ha, Sumapaz and Nevados amount to 180,000 ha and 45,000 ha, respectively. The high diversity registered for Frontino-Urrao may be due to the lack of significant habitat fragmentation by human development or agriculture. Additionally, numerous botanical explorations to the region have allowed for adequate knowledge of its diversity (Idárraga et al. 2011). According to Arias (2011), plant diversity of the Frontino-Urrao páramo is related to its wetlands and is propitiated in its diversity by the landscape heterogeneity represented therein.

The high diversity found in the Sonsón páramo is of great relevance, with 231 species being found in a very small area. This complex has great importance for the connection of the páramo biota of the Central Cordillera because it forms an intermediate point that could permit genetic exchange among the populations located in the Nevado del Ruiz peak and the Belmira páramo. It is worth mentioning that our explorations led to the discovery of a new species of *Espeletia* in the Sonsón páramo, which is in the process of being described and published. This finding will add to the high number of endemic *Espeletia* species in the páramos (Diazgranados 2012).

The similarity among páramo complexes, assessed through species and family composition by using methods of metrical distances, shows Belmira and Valle de Aburrá as the most similar (Fig. 2). These two páramos are located in the Central Cordillera, quite close to each other, and their current separation is due to anthropogenic influences rather than biogeographical vicariance.

The study of Londoño et al. (2014) presents the Farallones de Citará páramo as quite unlike the remaining páramos of Antioquia. In this analysis, we present a wider sampling of the diversity in Farallones de Citará (174 spp) that allowed us to compare its floral affinities more precisely. The work of Londoño et al. (2014) only considered 62 species for this páramo, probably causing separation of this complex from the remaining Antioquia páramos. We believe that this result is an effect of the low sampling used in the analysis. The páramos with low sampling effort in our study, Paramillo and Farallones de Citará, proved to be more dissimilar to the remaining ones when compared by families, genera, and species. Thus, it is possible that improving the knowledge of its flora might change biological affinities. Affinities remained the same when these analyses were repeated excluding these two areas.

The low diversity reported for the Farallones de Citará and Paramillo complex (Table 1) is likely a consequence of the scarce exploration that has been carried out, rather than being an accurate indicator of species composition (Idárraga et al. 2011). Thus, further studies should be conducted to expand the knowledge of biological diversity and conservation in these páramos. Although the páramo of Belmira is smaller than Farallones de Citará, it has greater diversity, possibly because more botanical expeditions have occurred in Belmira than Farallones de Citará.

The most diverse Angiosperm families in the páramos of Antioquia are the same as reported for other páramos of Colombia’s Eastern Cordillera, such as Chingaza (Vargas and Pedraza 2004, Madriñan 2012), Sumapaz (Franco and Betancur 1999), and the Podocarpus National Park in southern Ecuador (Lozano et al. 2009, Keating 1999). Asteraceae and Poaceae are the most diverse families in the Sierra Nevada de Mérida (Ricardi et al. 1997) and in the páramo of Ramal de Guaramacal in Venezuela, according to Cuello et al. (2010). In Colombia, these two families are the most diverse in the páramos of the Serranía del Perijá (Rivera-Díaz 2007) and in Chisacá (Pedraza et al. 2001).

The Asteraceae family has very high diversity in the explored páramos, especially in Frontino-Urrao, where it is represented by 59 species, almost 13% of the species of this páramo. With these values, Frontino-Urrao is a biodiversity hotspot for the Asteraceae family and constitutes an area of great interest for the study and conservation of this group, only comparable to ecosystems such as Chisacá, where 55 species for this family have been reported (Pedraza et al. 2004). An assessment of the floral composition for each of the explored páramos reveals that in Belmira, Farallones de Citará, and Sonsón, Melastomataceae is the family with the second highest diversity of species. Melastomataceae has been reported by Lozano et al. (2009) and Keating (1999) as the most diverse taxon of the Podocarpus Natural Park in the south of Ecuador.

*Epidendrum* L., *Miconia* Ruiz & Pav., and *Peperomia* Ruiz & Pav. with 21, 21, and 15 species, respectively, were the most diverse genera in the páramos of Antioquia. Both *Epidendrum* and *Miconia* provide a relevant contribution to páramo diversity in páramos such as Sumapaz (Franco and Betancur 1999), but the high diversity found in the páramos of Antioquia for *Peperomia* has not been previously reported for other páramo regions. Some genera found in the study such as *Aegiphila* Jacq., *Allophylus* L., *Minthostachys* (Benth.) Spach, *Polygala* L., *Ruagea* H. Karst., and *Styrax* L. have not been registered before as páramo flora components (Sklenar et al. 2005). Similarly, through this exploration we added 21 new records of species to the inventory of Antioquia flora.

These explorations allowed us to confirm the presence of species endemic to the páramos of Antioquia, such as *Diplostephium antioquense* Cuatrec., *Pentacalia sonsonensis* (Cuatrec.) Cuatrec., and *Pentacalia tomasiana* (Cuatrec.) Cuatrec. In our sampling, some rare species are outstanding too; we collected *Polygala corifolia* Planch. & Triana, a species only known from the type collection carried out in 1837 in the Sabana de Bogotá (Alzate 2016a), in the Belmira complex. Kattan et al. (2004) explained the occurrence of large numbers of endemic groups in páramos through geological history and the isolation of these ecosystems. Endemicity estimated for páramo plants is between 18% (Hofstede 2003) and 60% (Luteyn and Churchill 1999), considered as a whole ecosystem. There is not enough data to estimate endemicity rates of the páramos of Colombia because of missing information and taxonomical inconsistences for many taxa.

Our study confirms páramos as important habitats for threatened species. We found that 80 out of 693 species registered have some degree of vulnerability diagnosis from CITES or IUCN. These species are all members of Orchidaceae and Bromeliaceae, since these are the only families that have been subjects of conservation assesments (Garcia and Galeano 2006; Betancur and García 2006). Of the páramos in this study, Valle de Aburrá has the highest degree of human transformation, mainly due to urban expansion and the almost total destruction of original vegetation cover. Due to cattle ranching and mining very close to the forest-páramo boundary, Belmira has also been significantly altered by human activity. Due to its small and fragmented area, Sonsón is the most endagered páramo. This complex is composed of small areas on mountain peaks with agriculture threatening the remaining habitat. Meanwhile Urrao and Farallones de Citará are the best-preserved páramos of Antioquia, partly due to extreme topography and difficult access.

The inventory presented here is a detailed and wide addition to knowledge of Angiosperm diversity for a considerable extent of the páramos of Colombia and provides support for the high diversity of these ecosystems.
